# A comparative assessment of dry eye disease among outdoor street sweepers and indoor office cleaners

**DOI:** 10.1186/s12886-021-02025-y

**Published:** 2021-06-26

**Authors:** Chigozie I. Echieh, Bassey A. Etim, Chidiebere Peter Echieh, Taiwo Oyeniyi, Jeff Ajewole

**Affiliations:** 1 Department of Ophthalmology, University of Calabar Teaching Hospital, Calabar, Cross River State Nigeria; 2 Department of Ophthalmology, University of Calabar and University of Calabar Teaching Hospital, Calabar, Cross River State Nigeria; 3 Department of Surgery, University of Calabar, and University of Calabar Teaching Hospital, Calabar, Cross River State Nigeria; 4 Department of Community Medicine, University of Calabar Teaching Hospital, Calabar, Nigeria

**Keywords:** Dry eye disease, Ocular surface disorder, occupational health

## Abstract

**Background:**

Occupational predisposition to dry eye disease is known. Simultaneous exposure to multiple factors may pose more risk. Street sweepers are exposed to sunlight in addition to dust which all sweepers are exposed to. Tropical climate predisposes to significant exposure to sunlight. Combined exposure to dust and sunlight may lead to a synergy of factors. This study aims to assess the prevalence of dry eye disease (DED) amongst Street sweepers and Office cleaners in Calabar metropolis.

**Methods:**

A cross-sectional study was conducted among street sweepers and office cleaners. A systematic random sampling and multi-stage sampling method were used to select street sweepers (*n* = 115) and office cleaners (n = 115) respectively for the study. A pretested semi-structured interviewer-administered questionnaire was used to obtain information after which the respondents had an ophthalmic examination. An assessment of DED was done with Ocular Surface Disease Index (OSDI) questionnaire, Schirmer’s test, and tear break up time (TBUT). OSDI scores of 33 and above; Schirmer’s test readings of < 10 mm wetting in 5 min and a TBUT of less than 10s in either eye were considered as positive dry eye disease.

Results

The majority of respondents were females 215(93.5%) compared to males 15 (6.5%). The overall mean age of respondents was 40.96 ± 9.8 years. The average OSDI score, Schirmer’s test as well as TBUT among participants was 26.4 ± 16.0, 16.44 ± 9.52 mm, and 12.38 ± 4.53 s respectively.

The prevalence of DED among Street sweepers was 35.7% compared to 20% among office cleaners using the OSDI questionnaire (*p* = 0.352).

The prevalence of DED among street sweepers was 32.2% compared to 30.4% among Office cleaners using the Schirmer’s test. (*p* = 0.73) The TBUT reported a prevalence of 38.3% of DED among Street sweepers compared to 32.2% in office cleaners. (*p* = 0.48) Overall; the prevalence of dry eye disease among Street sweepers and office cleaners using OSDI score, Schirmers test, as well as TBUT were not statistically significant (> 0.05) Street Sweepers had higher odds of developing dry eye disease compared to office cleaners (OR = 2.085; C.I. =1.106–3.929; *p* = 0.02). Negative correlation coefficient was observed between TBUT and OSDI (r_s_ = − 0.102; *p* = 0.125). This was not statistically significant.

**Conclusion:**

Street sweepers had a higher prevalence of dry eye disease compared to office cleaners due to a higher risk of increased exposure to environmental factors such as dust, smoke, and sunlight. This effect is possibly due to a synergy of factors. Studies on dose-response are warranted.

## Background

The ocular surface is a sensitive structure that includes the conjunctiva and cornea bordered by the upper and lower lids and covered by a thin layer of tear film [1]. Its main function is to enable clear vision in an open eye by contributing more than two-thirds of the total refractive power of the eye [2]. All parts of this surface are directly exposed to the external environment and are thus vulnerable to potential environmental insults [3].

Ocular surface disorder (OSD), a broad term encompassing Dry Eye Disease (DED), blepharitis and meibomian gland dysfunction, allergic eye disease as well as chemical and thermal burns; may result in decreased visual function and poor performance of workers [4]. Impaired visual function is an important factor that can adversely affect job output and quality [5, 6]. For example, Nichols et al. reported that dry eye causes negligible absenteeism but markedly reduces workplace performance and that impairment of workplace performance is more closely related to dry eye symptoms than to clinical signs [5].

Dry eye disease is an ocular surface disease, characterized by a disorder of the tear film resulting from tear deficiency or excessive evaporation which causes damage to the inter- palpebral ocular surface and is associated with symptoms of ocular discomfort [7]. The prevalence of dry eye disease ranges from 5 to 35% worldwide; in Nigeria, it is at 19.2% based on the ocular surface disease index data [8, 9]. Environmental factors play an important role in the etiology of dry eye disease [10]. Surface toxicity, oxidative stress and, inflammation are the mechanisms implicated [11]. Occupational predisposition resulting from sunlight and dust exposure in outdoor workers as well as prolonged use of computers among professionals are risk factors for developing dry eye disease [12]. Most of these factors have been studied singly.

Street sweeping is an increasingly popular outdoor occupation in major cities in Nigeria. Their work entails daily cleaning of the roads and pavements with brooms; collection and disposal of wastes; this makes them chronically exposed to environmental elements such as dust, fumes. When such exposure occurs in the tropics, exposure to ultraviolet radiation may be significant.

We hypothesize that working outdoors in the presence of sunlight and dust (as typified by street sweepers in Calabar) may be associated with a greater prevalence of dry eyes compared with working indoors in the presence of dust but the absence of direct sunlight (as typified by indoor cleaners). The aim of this was to determine and compare the prevalence of dry eye disease among street sweepers who work outdoors and indoor office cleaners in Calabar metropolis, Nigeria.

## Methods

### Recruitment of participants

In this cross-sectional study, 230 respondents were involved in the study. A systematic random technique was used to recruit one hundred and fifteen (115) street sweepers from the Calabar Urban Development Authority (CUDA), an agency responsible for maintaining environmental sanitation in the metropolis. The sampling interval was calculated by dividing the number of the street sweepers on the nominal roll of CUDA (400) by the sample size of 115. This gave a sample interval of 3. The first study participant meeting the criteria for recruitment and consenting to participate was randomly selected from the first three numbers of the sampling frame (the street sweepers on the nominal roll of CUDA). Subsequently, every eligible third participant was selected until the desired sample size was obtained.

On the other hand, a multi-stage sampling technique was used to select 115 office cleaners. The first stage involved the selection of five ministries from twenty-eight ministries in the metropolis using a simple random technique by balloting; In the second stage, 23 office cleaners were selected respectively from the five ministries by balloting. Respondents were excluded from the study if they were using any ocular medication or were contact lens wearers.

### Ethical approval

Ethical approval was obtained from the Health Research Ethical Committee of the University of Calabar Teaching Hospital before commencing the study.

### Sample size determination

The sample size was determined using the formula for comparison of two proportions. The average prevalence of ocular surface disorders among exposed workers (P_1_ = 14.0%) and the average prevalence of ocular surface disorders among unexposed workers (P_2_ = 3.125%) from previous study [13] conducted in Benin, Nigeria was used in the calculation. To take care of attrition, 10% of the calculated sample size was added. One hundred and fifteen subjects were recruited into the indoor sweepers and outdoor sweepers arms respectively, totaling 230 subjects.

### Procedure

Written informed consent was obtained from the respondents and an interviewer-administered questionnaire (see supplementary file) was used to obtain data on socio-demographics, work history, ocular surface disease index and ocular symptoms. All respondents underwent an ocular examination where dry eye disease was assessed using Schirmer’s 1 test and, tear film break-up time. Schirmer’s 1 test value of 10 mm in 5 mins on Whatman’s filter paper no. 41, and a-Tear film break up time (TBUT) value of < 10 s was used as a cut-off mark for assessing dry eye disease.

Statistical analysis was performed using SPSS software version 20. A *P* value of < 0.05 was considered statistically significant. The prevalence odds ratio was used to study the strength of the association of occupational risk factors (street sweepers and office cleaners) with dry eye disease.

Outcome definitions for Dry eye disease in this study as shown below:

1. OSDI score - OSDI scores of 33 and above
2. Schirmer’s 1 test – A reading of less than 10 mm wetting of the Schirmer’s strip in 5 min
3. TBUT – Appearance of the first dry spot around the central cornea in less than 10s

## Results

Overall, 230 respondents participated in the study. Fifty percent (50.0%) were outdoor street sweepers while the remaining 50.0% were indoor office cleaners. Female: male ratio was 1:0.03. Over half of the respondents (51.3%) were aged 40 years and below with a mean age of 40 ± 9.6 years.

The mean age of the outside street sweepers was 41.78 ± 10.39 years while that of the office cleaners was 40.26 ± 9.46 years (Table 1). There was no statistically significant difference between the mean ages of the different groups of workers (t = 1.187, *p* = 0.155) Slightly less than one-third (31.7%) of the respondents had been educated up to primary school level.

**Table 1:** Socio-demographic characteristics of street sweepers and office cleaners in the study of dry eye disease amongst outdoor street sweepers and indoor office cleaners

Variables	Street Sweepers (***n*** = 115) Freq. (%)	Office Cleaners (n = 115) Freq. (%)	Total (***N*** = 230) Freq. (%)		p- value
**Age (years)**					
< =20	1 (0.9)	1 (0.9)	2 (0.9)		0.267
21–30	14 (12.2)	21 (18.3)	35 (15.2)		
31–40	44 (38.3)	37 (32.2)	81 (35.2)		
41–50	34 (29.6)	38 (33.0)	72 (31.3)		
51–60	19 (16.5)	18 (15.7)	37 (16.0)		
> 60	3 (2.6)	0 (0.0)	3 (1.3)		
**Mean Age** ± SD	41.78 ± 10.39	40.26 ± 9.46	40.96 ± 9.8 ±10.8		0.155
**Sex**					
Male	3 (2.6)	12 (10.4)	15 (6.5)		0.016*
Female	112 (97.4)	103 (89.6)	215 (93.5)		
Level of education					
None	3 (2.6)	1 (0.9)	4 (1.7)		< 0.047*
Primary	40 (34.8)	29 (25.2)	69 (30.0)		
Secondary	57 (49.6)	55 (47.8)	112 (48.7		
Tertiary	15 (13.0)	30 (26.1)	45 (19.6)		
Duration of years on the job					
1–5 years	46 (40.0)	45 (39.1)	93 (38.60)		< 0.001*
6–10 years	31 (27.0	32 (27.8)	69 (28.6)		
11–15 years	24 (20.9)	9 (7.8)	35 (14.5)		
16–20 years	13 (11.3)	10 (8.7)	25 (10.0)		
21–25 years	0 (0)	5 (4.3)			
26-30 years	1 (0.9)	9 (7.8)			
> 30 years	0 (0.0)	5 (4.3)	21 (8.3)		

### Prevalence of dry eye disease using OSDI, Schirmer’s test 1, TBUT

Overall, dry eye disease was found in 64 of the 230 respondents assessed, giving a prevalence of 27.8% using the ocular surface disease index. Results obtained from the OSDI ranged from 0 to 78, with a mean score of 26.4 ± 16.0.

Overall, the prevalence of dry eye disease assessed by Schirmer’s test was 31.3%. Out of 230 respondents, 72 had abnormal Schirmer’s test. Results obtained from Schirmer’s test ranged from 1 mm to 35 mm with a mean of 16.44 ± 9.52.

The overall prevalence of dry eye disease assessed using the TBUT among street sweepers and office cleaners was 35.2%. Eighty-one (81) respondents out of 230 had abnormal TBUT. Results obtained from the TBUT ranged between 2 s to 35 s, with a mean of 12.38 ± 4.53.

### Comparing the prevalence of dry eye disease in street sweepers and office cleaners

Comparing the two groups based on OSDI results, dry eye disease was found in 41 out of 115 street sweepers giving a prevalence of 35.7%. On the other hand, dry eye disease was found in 23 out of 115 office cleaners resulting in a prevalence of 20%. This difference was not statistically significant (*p*-value 0.352).

Comparing the prevalence of dry eye disease diagnosed using Schirmer’s test in street sweepers and office cleaners showed that 37(32.2%) out of 115 street sweepers had abnormal Schirmer’s test compared to 35 (30.4%) of 115 office cleaners. The prevalence was higher in street sweepers compared to office cleaners. However, this difference was not statistically significant (*p*-value = 0.73).

Assessment of dry eye disease using TBUT showed that, out of 115 street sweepers, 44 had abnormal TBUT giving a prevalence of 38.3%. On the other hand, of the 115 office cleaners, 37 had abnormal TBUT giving a prevalence of 32.2%. The average TBUT in office cleaners was 12.88 ± 5.08 while the average TBUT among the street sweepers was 11.87 ± 3.83. This difference in mean TBUT was not statistically significant. (*p* = 0.09).

### Relationship between risk factors and the occurrence of dry eye disease defined by OSDI

Logistic regression was performed to assess the impact of statistically significant factors on the likelihood of developing dry eye disease. As shown in Table 2, the class of workers and ages of respondents had a statistically significant contribution to the occurrence of dry eye disease.

**Table 2:** Association of Dry eye (diagnosed by OSDI) with study groups

Variable	Dry Eye disease present		Dry Eye disease absent		Total		Chi square	p-value
	N	(%)	N	(%)	n	(%)		
**Group of Respondents**								
Outdoor Street sweepers	41	35.7	74	64.3	115	100	7.014	0.008*
Indoor office cleaners	23	20.0	92	80.0	115	100		

The result demonstrates that street sweepers were more likely to develop a dry eye disease compared to office sweepers with an odds ratio of 2.085 (Table 3). Another predictor observed in this study for developing dry eye disease was the age of respondents in years with a prevalence odds ratio of 1.092. This indicates that the increasing age of respondents is a weak predictor of the occurrence of dry eye disease.

**Table 3:** Logistic regression showing association of Age of Respondents with the occurrence of dry eye disease (diagnosed by OSDI)

Variable	Odds ratio	95% confidence Interval	p-value
**Study Group**			
Office cleaners	1		
Street sweepers	2.085	**1.106–3.929**	0.023*
**Age of respondents**	1.092	**1.054–1.132**	0.000*

* Statistically significant

*p* < 0.05

### Distribution of ocular complaints among workers

Table 4 shows the distribution of ocular complaints among respondents. The following ocular complaints had higher prevalence and were statistically significant in street sweepers compared to office cleaners; itching, tearing, redness of the eyes, foreign body sensation and, mucoid discharge.

**Table 4:** Distribution of ocular complaints among workers

Variables	Street Sweepers		Office Cleaners		p-Value
	Freq. (n = 115) (%)		Freq. (n = 115)	(%)	
**Itching**					
Yes	88 (76.5)		64 (55.7)		0.001*
No	27 (23.5)		51 (44.3)		
**Tearing**					
Yes	70 (60.9)		33 (28.7)		< 0.001*
No	45 (39.1)		82 (71.3)		
**Redness**					
Yes	54 (47.0)		19 (16.5)		< 0.001*
No	61 (53.0)		96 (83.5)		
**Foreign body sensation**					
Yes	42 (36.5)		24 (20.8)		0.007*
No	73 (63.5)		91 (79.2)		
**Mucoid discharge**					
Yes	25 (21.7)		13 (11.3)		0.033*
No	90 (78.2)		102 (88.7)		
**Gritty Sensation**					
Yes	19 (16.5)		11 (9.6)		0.122
No	96 (83.5)		104 (90.4)		
**Pains in the eyes whemn looking at light**					
Yes	12 (10.4)		4 (3.5)		0.897
No	103 (89.6)		yyyyy (96.5)		

*statistically significant

P < 0.05

### Relationship between ocular surface disease index (OSDI) and tear break up time (TBUT)

Table 5 describes the correlation between the OSDI and TBUT of the respondents. A negative correlation coefficient was observed for OSDI and TBUT which was not statistically significant. The negative correlation coefficient meant that as the tear breakup time results increased, the Ocular Surface Disease Index score decreased or revealed fewer symptoms.

**Table 5:** Correlation of OSDI with TBUT

Variables	r_s_	P-value	Significance
Tear Break up time(TBUT)	−0.182	0.007*	(Significant)
Ocular Surface Disease Index(OSDI)	1.000		

*Correlation is significant at 0.05 level (2-tailed)

## Discussion

Documented prevalence of dry eye disease among workers is diverse, this study sought to determine the prevalence of dry eye disease among street sweepers who are outdoor workers and office cleaners who work predominantly indoors. Both populations work in dusty environments, however street sweepers; in addition, are exposed to direct sunlight.

The prevalence of dry eye disease assessed subjectively using the ocular surface disease index questionnaire (OSDI) among all respondents was 27.8%. In addition, this study noted a prevalence of 31.4 and 35.3% respectively based on Schirmer’s test 1 and tear break up time (TBUT) respectively. This study showed a higher prevalence of dry eye disease in street sweepers compared to office cleaners with varied clinical tests. This finding could be explained by the fact that street sweepers are slightly more symptomatic as a result of continuous and long-term sweeping which exposes them more to environmental irritants such as dust and smoke. The prevalence rate in this study is higher than that reported in southeast Nigeria (19.2%) using the OSDI questionnaire [9]. The results of this study are comparable with other studies conducted among outdoor workers where the prevalence of dry eye disease was higher amongst outdoor workers compared with indoor workers [14, 15]. Our study showed a higher prevalence of dry eye disease in Street sweepers compared to office cleaners using the OSDI, Schirmer’s test, and Tear Break up time respectively. However, this was not statistically significant (*p* > 0.05). Suchi et al. reported a prevalence of 59.3% of dry eyes among outdoor jobs consisting of farmers and laborers and a prevalence of 42.6% in indoor jobs [14]. Khurana et al., reported an increased risk of dry eye among farmers and laborers (32 and 28%) respectively and attributed this increase to excessive exposure of these workers to adverse environmental conditions [15].

The ocular complaints among participants in this study were itching, redness of the eyes, foreign body sensation, and tearing, ocular pain, and mucoid discharge. (Table 4) This may result from a disturbance of the ocular surface where sand dust acts as direct irritants to the eyes. This corroborates with the study performed in Australia by McCarty et al. who reported that most of the patients with dry eyes presented with the symptoms of foreign body sensation, itching tearing and, photophobia [16].

The study noted that Street sweepers were twice more likely to develop dry eye disease compared to office cleaners. (OR = 2.085; 95% C. I = 1.106–3.929; *p* = 0.02) (Table 3) Furthermore, dry eye disease was noted to be associated with the age of the workers, with increasing age, a worker was more likely to develop dry eye disease. (OR = 1.092; 95% C. I = 1.054–1.132; *p* = 0.00) (Table 2). This finding corroborates an earlier study that noted dry eye disease to be significantly associated with increasing age [17].

The findings from our study further highlight the increased risk of developing dry eye disease in outdoor workers (street sweepers) constantly exposed to combined environmental irritants like dust and sunlight. The increased burden on street sweepers is likely to be due to the synergistic effect of irritants. The burden of DED among indoor cleaners may be attributed to working in an air-conditioned environment or to the use of cleaning sprays during work. A dose relationship between the burden of dry eyes and exposure to an irritant warrants further studies. Earlier studies on this subject [18] documented a high level of awareness on ocular health safety among street sweepers however, there was poor utilization of personal protective devices. Hence, we recommend improved awareness and strict use of protective gear. We recommend legislative prescription of adherence to the use of sunscreens and dust-proof hoods that will reduce the exposure of street sweepers to multiple occupational hazards. We also suggest that outdoor sweeping could be scheduled to take place during the evening hours when the exposure to sunlight is minimal.

### Limitation of study

It was not possible, during this study, to standardize the exposure to dust in both groups. However, both groups are involved in sweeping, which raises dust. Also, the interviewers/test personnel were not blinded to the test subjects’ occupation. This was because the authors had to conduct the interview and do the dry eye tests themselves due to the limitation of resources. This may have introduced bias. However, standardization of the tests was done prior to the commencement and during the study. Specifically, TBUT was done three times and the mean of the result was used for the analysis.

## Conclusion

This study concluded that **s**treet sweepers had higher odds of developing dry eye disease compared to office cleaners. This effect is possibly due to the synergy of environmental factors. Studies on dose-response are warranted.

## Data Availability

The datasets used and/or analyzed during the current study are available from the corresponding author on reasonable request.

## Abbreviations

DED: Dry eye disease
OSDI: Ocular surface disease index
TBUT: Tear break up time
CUDA: Calabar Urban Development Authority
